# Potential use of transcranial sonography in the sick patient

**DOI:** 10.1186/cc9742

**Published:** 2011-03-11

**Authors:** G Hadjipavlou, O Touma

**Affiliations:** 1 Nuffield Department of Anaesthetics, Oxford, UK; 2 Oxford Radcliffe Hospitals, Oxford, UK

## Introduction

Transcranial sonography (TCS) is used to image brain parenchyma and vasculature. There is a growing body of evidence suggesting a possible imaging role and that Doppler reflects intra-cranial pressure. The authors conducted a review of this growing literature and propose potential uses of this modality in the assessment of the sick patient.

## Methods

A search for papers of special interest was conducted using PubMed and the search items: transcranial, ultrasound, sonography, raised intracranial pressure, haemorrhage, and traumatic head injury. Articles where restricted to adults and considered relevant if they described standardisation, comparisons with other modalities, case studies or explored potential novel uses.

## Results

TCS has been standardised and referenced to MRI imaging. It is able to identify intracerebral, and subarachnoid haemorrhage as areas of hyperechogenicity. Compared with CT, it identifies haemorrhage or infarct in 95% of cases. TCS is a reliable quantitative monitor of intracranial pressure. The pulsatility index (PI), a derived index from Doppler flow parameters of the middle cerebral artery, correlates significantly with invasive measures of intracranial pressure (ICP); *R *= 0.98, *P *< 0.001. A formula can be used to convert the PI into ICP.

## Conclusions

TCS has imaging potential, but is unlikely to replace CT for this purpose. The role for TCS in the assessment and monitoring of the sick patient starts where CT fails. It can be used as a quick screening adjunct to the primary survey looking for acute brain injury in those unstable for transfer. It can be used to monitor the size of CT-identified haemorrhage over time or with GCS removing the need for multiple trips to the scanner. It could help identify raised ICP and therefore extra risk from lumbar puncture in the meningitic patient with a normal CT. Finally it allows non-invasive monitoring of ICP in the head-injured patient in whom intubation and sedation are required, but invasive monitoring would be considered excessive.

## References

[B1] Caricato A Intensive Care Med 2010 36 1091 1092 10.1007/s00134-010-1801-0 20213067

[B2] Czosnyka M J Neurosurg 1998 88 802 808 10.3171/jns.1998.88.5.0802 9576246

[B3] Bellner J Surg Neurol 2004 62 45 51 10.1016/j.surneu.2003.12.007 15226070

[B4] Kern R Ultrasound Med Biol 2005 31 311 315 10.1016/j.ultrasmedbio.2004.12.006 15749552

[B5] Maurer M Stroke 1998 29 2563 2567 9836768 10.1161/01.str.29.12.2563

[B6] Meyer-Wiethe K Cerebrovasc Dis 2009 27 Suppl 2 40 47 10.1159/000203125 19372659

